# Diagnostic validity and solute-corrected prevalence for hyponatremia and hypernatremia among 1 813 356 admissions

**DOI:** 10.1093/ckj/sfae319

**Published:** 2024-10-24

**Authors:** Akira Okada, Hayato Yamana, Hideaki Watanabe, Katsunori Manaka, Sachiko Ono, Kayo Ikeda Kurakawa, Masako Nishikawa, Makoto Kurano, Reiko Inoue, Hideo Yasunaga, Toshimasa Yamauchi, Takashi Kadowaki, Satoko Yamaguchi, Masaomi Nangaku

**Affiliations:** Department of Prevention of Diabetes and Lifestyle-Related Diseases, Graduate School of Medicine, The University of Tokyo, Tokyo, Japan; Data Science Center, Jichi Medical University, Shimotsuke, Japan; Department of Clinical Epidemiology and Health Economics, The University of Tokyo, Tokyo, Japan; Division of Nephrology and Endocrinology, Graduate School of Medicine, The University of Tokyo, Tokyo, Japan; Department of Eat-loss Medicine, Graduate School of Medicine, The University of Tokyo, Tokyo, Japan; Department of Prevention of Diabetes and Lifestyle-Related Diseases, Graduate School of Medicine, The University of Tokyo, Tokyo, Japan; Department of Clinical Laboratory, University of Tokyo Hospital, Tokyo, Japan; Department of Clinical Laboratory, University of Tokyo Hospital, Tokyo, Japan; Department of Clinical Laboratory Medicine, Graduate School of Medicine, The University of Tokyo, Tokyo, Japan; Department of Prevention of Diabetes and Lifestyle-Related Diseases, Graduate School of Medicine, The University of Tokyo, Tokyo, Japan; Department of Clinical Epidemiology and Health Economics, The University of Tokyo, Tokyo, Japan; Department of Diabetes and Metabolism, Graduate School of Medicine, The University of Tokyo, Tokyo, Japan; Department of Prevention of Diabetes and Lifestyle-Related Diseases, Graduate School of Medicine, The University of Tokyo, Tokyo, Japan; Department of Diabetes and Metabolism, Graduate School of Medicine, The University of Tokyo, Tokyo, Japan; Toranomon Hospital, Tokyo, Japan; Department of Prevention of Diabetes and Lifestyle-Related Diseases, Graduate School of Medicine, The University of Tokyo, Tokyo, Japan; Division of Nephrology and Endocrinology, Graduate School of Medicine, The University of Tokyo, Tokyo, Japan

**Keywords:** clinical epidemiology, database study, electrolytes, solute correction, validation study

## Abstract

**Background and hypothesis:**

We aimed to evaluate the diagnostic validity of the International Classification of Diseases, 10th Revision (ICD-10) codes for hyponatremia and hypernatremia, using a database containing laboratory data. We also aimed to clarify whether corrections for blood glucose, triglyceride, and total protein may affect the prevalence and the diagnostic validity.

**Methods:**

We retrospectively identified admissions with laboratory values using a Japanese hospital-based database. We calculated the sensitivity, specificity, and positive/negative predictive values of recorded ICD-10-based diagnoses of hyponatremia (E87.1) and hypernatremia (E87.2), using serum sodium measurements during hospitalization (<135 and >145 mmol/l, respectively) as the reference standard. We also performed analyses with corrections of sodium concentrations for blood glucose, triglyceride, and total protein.

**Results:**

We identified 1 813 356 hospitalizations, including 419 470 hyponatremic and 132 563 hypernatremic cases based on laboratory measurements, and 18 378 hyponatremic and 2950 hypernatremic cases based on ICD-10 codes. The sensitivity, specificity, positive predictive value, and negative predictive value of the ICD-10 codes were 4.1%, 99.9%, 92.5%, and 77.6%, respectively, for hyponatremia and 2.2%, >99.9%, 96.5%, and 92.8%, respectively, for hypernatremia. Corrections for blood glucose, triglyceride, and total protein did not largely alter diagnostic values, although prevalence changed especially after corrections for blood glucose and total protein.

**Conclusions:**

The ICD-10 diagnostic codes showed low sensitivity, high specificity, and high positive predictive value for identifying hyponatremia and hypernatremia. Corrections for glucose or total protein did not affect diagnostic values but would be necessary for accurate prevalence calculation.

## Graphical Abstract

**Graphical Abstract:**
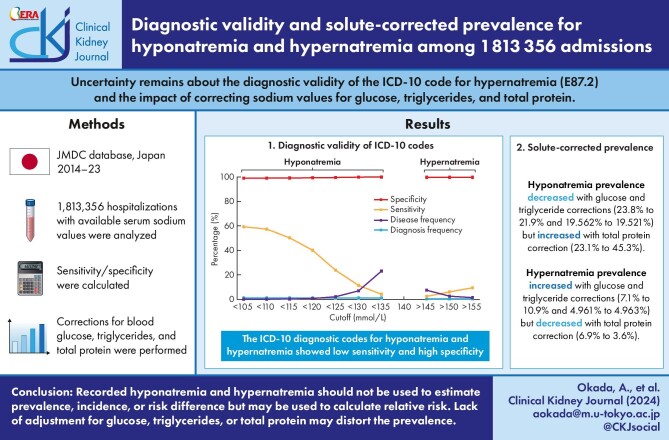

KEY LEARNING POINTS **What was known**:

- Previous articles have shown that the validity of the International Classification of Diseases, 10th Revision (ICD-10) code for hyponatremia (E87.1) had a low sensitivity and high specificity, but that for hypernatremia (E87.2) has never been evaluated.
- In most current laboratory measurements (using indirect ion-specific electrodes), sodium concentrations are affected by blood glucose, triglycerides, and total protein.

**This study adds**:

- The ICD-10 diagnostic code for hypernatremia showed low sensitivity, high specificity, and high positive predictive value.
- Corrections for glucose or total protein did not affect diagnostic values for the ICD-10 codes but affected the prevalence of hyponatremia and hypernatremia.

**Potential impact**:

- Recorded hyponatremia and hypernatremia should not be used to calculate the prevalence, incidence, or risk difference but may be used to calculate a relative risk.
- The failure to adjust for values of blood glucose, triglycerides, or total protein may underestimate or overestimate the prevalence of both sodium disorders.

## INTRODUCTION

Epidemiological studies using large-scale administrative databases have been attracting worldwide attention in recent years [1]. Administrative databases enable researchers to conduct large-scale studies in real-world settings [2]. However, such databases are used under the assumption that they convey accurate information on health conditions and service provision [3]. Validating the data stored in administrative databases is crucial because the misclassification of exposures or outcomes may lead to biased results when performing database studies [4]. Previous database studies using diagnostic codes for hyponatremia have reported the validity of code-based diagnosis [5, 6].

Several validation studies on hyponatremia have been conducted in different countries [7–10]. A study on the International Classification of Diseases, Ninth Revision, Clinical Modification diagnosis codes for hyponatremia in an outpatient setting revealed a sensitivity of 3.5% and specificity of >99% for identifying hyponatremia (serum sodium <136 mmol/l) [7]. A study on hyponatremia in inpatients reported a sensitivity of 1.7% and a specificity of >99% [8]. The International Classification of Diseases, Tenth Revision (ICD-10) codes for hyponatremia have been reported to have a similarly low sensitivity and high specificity [9, 10].

Evidence is lacking on validation studies of the ICD-10 codes for hypernatremia, another important electrolyte abnormality. Furthermore, most current laboratory measurement systems use indirect ion-specific electrodes [11], with which the presence of high levels of solutes such as blood glucose, triglycerides, and total protein [12–14] can disrupt the correlation between sodium concentration and plasma tonicity, leading to inaccurate assessments of osmolality [11, 15]. In particular, severe hypertriglyceridemia or paraproteinemia can cause measurement artifacts in the dilution process of indirect ion-specific electrodes, which may lead to an underestimation or overestimation of tonicity-based sodium disorders [16]. Although a few articles reported solute-corrected prevalence of hyponatremia [17, 18], possible effects of the corrections on the prevalence of hyponatremia and hypernatremia have not been discussed in general hospitalizations.

This study examined the accuracy of diagnoses of hyponatremia and hypernatremia in hospitalizations using a hospital-based database. We also evaluated the possible effects of these corrections on the prevalence of hyponatremia and hypernatremia.

## MATERIALS AND METHODS

### Data source

We used data from the JMDC hospital-based database (JMDC Inc., Tokyo, Japan). The details of this database have been described previously [19]. Briefly, the database contains the Japanese Diagnosis Procedure Combination (DPC) data, claims data, and laboratory values obtained from ∼95 hospitals. The Japanese government introduced the DPC system to standardize the electronic claims system and to realize transparency of hospital performance [20]. The DPC data of the JMDC database are recorded in a manner similar to that stored in other databases and include the following items: patient demographics, detailed clinical information on diseases, patient statuses at admission and discharge, diagnoses, procedures, and medications. Diagnoses are recorded based on ICD-10 codes and Japanese free text, recorded by the attending physicians [21]. Suspected diagnoses are recorded and are denoted accordingly. Six categories of diagnoses exist: “main diagnosis,” “admission-precipitating diagnosis,” “comorbidities at admission,” “complications occurring after admission,” “most resource-consuming diagnosis,” and “second most resource-consuming diagnosis” [20]. The DPC data have a distinctive property in that the main diagnosis, comorbidities at admission, and complications during hospitalization are clearly distinguishable among the recorded diagnoses [20, 21]. An increasing number of validation studies on procedure codes and disease names in the DPC data have been published [22–26].

### Study population

Using the JMDC database, we identified patients aged ≥18 years who were discharged between 1 April 2014 and 31 August 2023 and had at least one measurement for serum sodium concentration during hospitalization. The exclusion criteria were as follows: absence of information on age, sex, whether the admission was unscheduled, ambulance use, consciousness level on admission, or admission-precipitating diagnosis and maximum sodium concentration of >230 mmol/l during hospitalization, and minimum sodium concentration of <90 mmol/l during hospitalization. The criteria for erroneous values were based on case reports of hypernatremia (209 mmol/l) [27] and hyponatremia (98 mmol/l) [28], with an additional 10% margins. Repeated hospitalizations for a single patient were included and analyzed independently.

### Study outcomes and variables

The following patient information was extracted from the database: age; sex; body mass index (BMI); main diagnosis, admission-precipitating diagnosis, comorbidities at admission, and complications occurring after admission; smoking status (current/past or non-smoker); consciousness on admission according to the Japan Coma Scale; activities of daily living according to the Barthel index [29], and in-hospital death.

We extracted all serum sodium concentration test results during hospitalization and summarized them as daily data. We primarily defined hyponatremia as a serum sodium concentration of <135 mmol/l [10, 30–32] and also performed analyses with <130 and <125 mmol/l for moderate and severe hyponatremia, respectively [32]. Hypernatremia was defined as a serum sodium concentration of >145 mmol/l [33–35], as well as >150 and >155 mmol/l for moderate and severe hypernatremia, respectively. In cases where a patient underwent multiple examinations on a single day, the most extreme measurements were recorded.

The ICD-10 codes of E87.1 and E87.2 were used to identify hyponatremia and hypernatremia from the DPC data, respectively. We categorized each hospitalization based on the ICD-10 code of the admission-precipitating diagnosis as follows: infectious (A00–B99), neoplasms (C00–D48), hematological (D50–D89), endocrinological (E00–E90), mental (F00–F99), neurological (G00–G99), ophthalmological (H00–H59), otological (H60–H95), cardiovascular (I00–I99), respiratory (J00–J99), digestive (K00–K93), dermatological (L00–L99), musculoskeletal (M00–M99), genitourinary (N00–N99), pregnancy (O00–O99), perinatal (P00–P96), congenital (Q00–Q99), symptoms/signs (R00–R99), injury/poisoning (S00–T98), new diseases (U00–U99), external causes (V00–Y98), and examinations (Z00–Z99).

The Institutional Review Board of the Graduate School of Medicine of The University of Tokyo (2018030NI) approved the study protocol. Owing to the anonymous nature of the data, the requirement for informed consent was waived.

### Statistical analysis

We first summarized the background characteristics of the eligible population. We subsequently categorized individuals into two groups based on the occurrence of hyponatremia (<135 mmol/l) during hospitalization. We compared the distributions between those with and without hyponatremia using the Wilcoxon rank-sum test for continuous variables and *χ^2^* tests for categorical variables. Similarly, we classified the eligible individuals into two groups based on the occurrence of hypernatremia (>145 mmol/l) during hospitalization and compared their characteristics. We also identified the 10 most frequent admission-precipitating diagnoses among all the patients, those with hyponatremia and those with hypernatremia, based on the first three digits of the ICD-10 codes.

**Figure 1:**
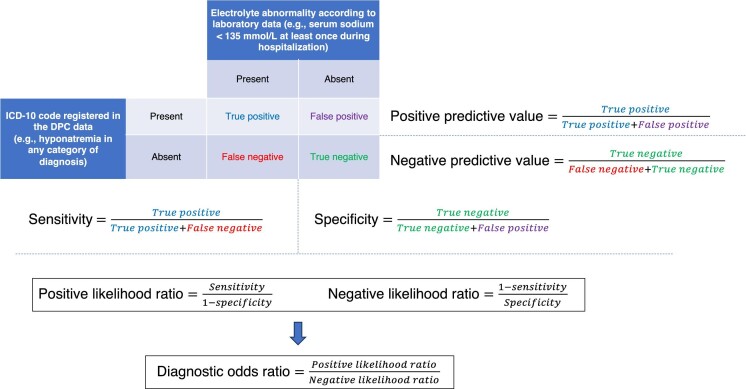
Definition of validity indices in this study.

Using laboratory data as the reference standard, we examined the validity of DPC data for identifying hyponatremia in three different scenarios corresponding to the timing of diagnosis: using all six categories of diagnoses to identify hyponatremia observed at least once during hospitalization, using diagnoses present at the time of admission (i.e. “admission-precipitating diagnosis” or “comorbidities at admission”) to identify hyponatremia observed on the day of admission, and using diagnoses occurring after admission (i.e. “complications occurring after admission”) to identify hyponatremia observed at least once on the second day of hospitalization or later. For the last two scenarios, those with no measured sodium levels at the respective time points were excluded from the analyses. We calculated the sensitivity, specificity, positive likelihood ratio (LR+), negative likelihood ratio (LR−), diagnostic odds ratio (DOR), positive predictive value (PPV), and negative predictive value (NPV) of DPC data-based identification against the reference thresholds of <135, <130, and <125 mmol/l serum sodium. These procedures were repeated for hypernatremia, with the reference thresholds of >145, >150, and >155 mmol/l. An overview of the validity indices in this study is shown in Fig. 1. We also performed analyses stratified by age (the median of eligible individuals) and the admission-precipitating disease (neoplasms, cardiovascular, respiratory, and others). As a supplementary analysis, we used 120, 115, 110, and 105 mmol/l as cutoffs for hyponatremia. We provided the statistics for these cutoffs because sodium levels of ≥120 mmol/l are considered a safe range for developing osmotic demyelination syndrome [36] , while sodium levels of <105 mmol/l suggest the highest-risk group for developing osmotic demyelination syndrome [37]. We performed two sensitivity analyses: one limiting hospitalizations to the first one during the research period for each patient, and the other excluding individuals with diabetes (ICD codes E10–E14), hypertriglyceridemia (E78.1), or multiple myeloma (C90.0).

Considering that blood glucose, triglyceride, and total protein levels reportedly affect sodium concentrations [12–14], we calculated statistics before and after correction for these values among individuals with data for all these parameters. Marked hyperglycemia [11, 15], hypertriglyceridemia or paraproteinemia [16], and hypoproteinemia [17] can disrupt the correlation between sodium concentration and plasma tonicity. We used laboratory values for these three items, which were measured on the same day as the sodium measurement of interest. In cases where a patient underwent multiple examinations on a single day, the most extreme values for glucose, triglycerides, and total protein were recorded. We corrected sodium concentrations as follows [12–15]:


\begin{eqnarray*}{\mathrm{Glucose}} &-& {\mathrm{corrected\ sodium\ concentration\ }} \nonumber\\
&=& {\mathrm{\ }}\left[ {{\mathrm{N}}{{{\mathrm{a}}}^ + }} \right]{\mathrm{\ }} + {\mathrm{\ }}2.4 \times \frac{{\textit{glucose}\ - 100}}{{100\ }}\end{eqnarray*}

\begin{eqnarray*}{\mathrm{TG}} &-& {\mathrm{corrected\ sodium\ concentration\ }}\nonumber\\
&=& {\mathrm{\ }}\left[ {{\mathrm{N}}{{{\mathrm{a}}}^ + }\left] {{\mathrm{\ }} + } \right[{\mathrm{N}}{{{\mathrm{a}}}^ + }} \right]{\mathrm{\ }} \times \frac{{2.1 \times TG - 0.6}}{{100}}\end{eqnarray*}

\begin{eqnarray*}{\mathrm{TP}} &-& {\mathrm{corrected\ sodium\ concentration\ }}\nonumber\\
&=& {\mathrm{\ }}\left[ {{\mathrm{N}}{{{\mathrm{a}}}^ + }} \right]{\mathrm{\ }} \times \frac{{93}}{{99.1 - 0.7 \times TP}}\end{eqnarray*}

where [Na^+^] indicates measured sodium concentration, glucose refers to blood glucose concentration (mg/dl), TG refers to triglyceride concentration (g/l), and TP stands for total protein level (g/dl) (equations for different units are shown in the Supplementary Methods).

The linear function was used for glucose-corrected and total-protein-corrected sodium concentrations across all ranges of each value, while the cubic function was applied for triglyceride-corrected sodium concentrations exceeding 1500 mg/dl, according to previously described methods [12–15].

Because of the large sample sizes, we did not perform statistical testing. Stata version 18 (StataCorp, College Station, TX, USA) was used to conduct all statistical analyses.

## RESULTS

### Study population

**Figure 2:**
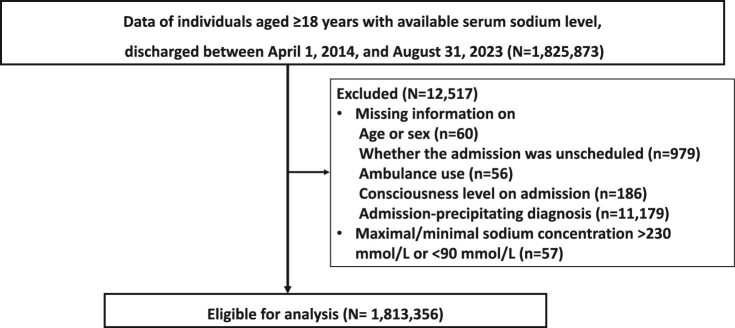
Flow chart for the included patients.

We extracted data from 1 825 873 hospitalizations with recorded serum sodium levels in the JMDC database. After excluding 12 517, we obtained 1 813 356 records from 1 156 291 patients for analysis (Fig. 2). We identified 419 470 (23.1%) and 132 563 (7.3%) hospitalizations with hyponatremia (serum sodium level of <135 mmol/l) and hypernatremia (serum sodium level of >145 mmol/l), respectively. Conversely, 18 378 (1.0%) and 2950 (0.2%) had ICD-10-based hyponatremia and ICD-10-based hypernatremia, respectively. Patients with hyponatremia (<135 mmol/l) were more likely to be older, leaner, exhibit disturbed consciousness, and die during hospitalization than those without hyponatremia (Table 1). These characteristics were also observed in patients with hypernatremia based on sodium levels (Table 2). However, while the proportion of males was higher in the group with hyponatremia (<135 mmol/l) compared with the group without, the hypernatremia group (>145 mmol/l) had a higher proportion of females than the non-hypernatremia group. The most frequent admission-precipitating diagnosis among all hospitalizations was heart failure (ICD-10, I50), followed by cerebral infarction (I63) and angina pectoris (I20), as shown in Table 3. Among patients with hyponatremia (<135 mmol/l) and hypernatremia (>145 mmol/l), the most frequent admission-precipitating diagnosis was heart failure (ICD-10, I50), followed by pneumonitis due to solids and liquids (J69).

**Table 1:** Characteristics of eligible individuals with and without hyponatremia as evidenced by recorded serum sodium levels.

		Total	Not hyponatremic during hospitalization	Hyponatremic during hospitalization
Variable	Category	*N* = 1 813 356	*N* = 1 393 886	*N* = 419 470
Age category	18–49 years	233 118 (12.9)	207 197 (14.9)	25 921 (6.2)
	50–64 years	269 105 (14.8)	224 005 (16.1)	45 100 (10.8)
	65–79 years	657 411 (36.3)	509 665 (36.6)	147 746 (35.2)
	≥80 years	653 722 (36.1)	453 019 (32.5)	200 703 (47.8)
Male		953 378 (52.6)	717 572 (51.5)	235 806 (56.2)
Minimum sodium concentration on admission day (mmol/l)		139 [136–141]	140 [138–142]	134 [131–137]
Minimum sodium concentration during hospitalization (mmol/l)		138 [135–140]	139 [137–141]	132 [129–133]
Minimum sodium concentration after admission day (mmol/l)		138 [135–140]	139 [137–141]	132 [130–134]
Maximum sodium concentration on admission day (mmol/l)		139 [137–141]	140 [138–142]	134 [132–138]
Maximum sodium concentration during hospitalization (mmol/l)		141 [139–143]	141 [140–143]	139 [136–141]
Maximum sodium concentration after admission day (mmol/l)		141 [139–143]	141 [140–143]	139 [136–141]
Number of days with sodium measurement per week		2.0 [1.2–3.0]	2.0 [1.2–2.9]	2.2 [1.4–3.0]
Admission-precipitating diagnosis	Infectious disease	40 562 (2.2)	28 302 (2.0)	12 260 (2.9)
based on ICD-10 code	Malignancy	335 296 (18.5)	258 669 (18.6)	76 627 (18.3)
	Blood disorders	15 052 (0.8)	10 451 (0.7)	4601 (1.1)
	Endocrinological	65 466 (3.6)	42 244 (3.0)	23 222 (5.5)
	Mental disorders	7785 (0.4)	6790 (0.5)	995 (0.2)
	Neurological	43 666 (2.4)	36 206 (2.6)	7460 (1.8)
	Ophthalmological	5791 (0.3)	5481 (0.4)	310 (0.1)
	Otological	16 750 (0.9)	15 902 (1.1)	848 (0.2)
	Cardiovascular	361 762 (19.9)	286 766 (20.6)	74 996 (17.9)
	Respiratory	149 954 (8.3)	96 660 (6.9)	53 294 (12.7)
	Digestive	237 913 (13.1)	182 506 (13.1)	55 407 (13.2)
	Dermatological	18 510 (1.0)	13 323 (1.0)	5187 (1.2)
	Musculoskeletal	90 913 (5.0)	77 695 (5.6)	13 218 (3.2)
	Genitourinary	121 383 (6.7)	90 617 (6.5)	30 766 (7.3)
	Pregnancy	39 033 (2.2)	33 086 (2.4)	5947 (1.4)
	Perinatal	50 (0.0)	41 (0.0)	9 (0.0)
	Congenital	2866 (0.2)	2552 (0.2)	314 (0.1)
	Symptoms/signs	21 432 (1.2)	15 391 (1.1)	6041 (1.4)
	Injury/poisoning	200 981 (11.1)	164 344 (11.8)	36 637 (8.7)
	New diseases	36 591 (2.0)	25 543 (1.8)	11 048 (2.6)
	External causes	4 (0.0)	3 (0.0)	1 (0.0)
	Examinations	1596 (0.1)	1314 (0.1)	282 (0.1)
BMI (kg/m^2^)		22.2 [19.9–24.6]	22.4 [20.1–24.8]	21.2 [19.0–23.7]
BMI category	<18.5 kg/m^2^	191 684 (10.6)	127 515 (9.1)	64 169 (15.3)
	18.5–<25 kg/m^2^	943 232 (52.0)	736 387 (52.8)	206 845 (49.3)
	≥25 kg/m^2^	325 582 (18.0)	274 479 (19.7)	51 103 (12.2)
	Missing	352 858 (19.5)	255 505 (18.3)	97 353 (23.2)
Smoking history	Non-smoker	1 150 678 (63.5)	886 579 (63.6)	264 099 (63.0)
	Current/past smoker	460 159 (25.4)	358 062 (25.7)	102 097 (24.3)
	Missing	202 519 (11.2)	149 245 (10.7)	53 274 (12.7)
Consciousness level	Alert	1 548 278 (85.4)	1 222 968 (87.7)	325 310 (77.6)
	Not clear	265 078 (14.6)	170 918 (12.3)	94 160 (22.4)
Charlson comorbidity index		0 [0–2]	0 [0–1]	0 [0–2]
Activities of daily living	Independent	971 959 (53.6)	823 292 (59.1)	148 667 (35.4)
	Dependent	700 965 (38.7)	472 774 (33.9)	228 191 (54.4)
	Missing	140 432 (7.7)	97 820 (7.0)	42 612 (10.2)
Unscheduled admission		1 087 416 (60.0)	772 877 (55.4)	314 539 (75.0)
Ambulance use		502 607 (27.7)	348 248 (25.0)	154 359 (36.8)
In-hospital death		112 226 (6.2)	54 680 (3.9)	57 546 (13.7)

Data are presented *N* (%) for categorical variables and median [1st–3rd quartile] for continuous variables.

BMI, body mass index; ICD-10, the International Classification of Diseases, Tenth Revision

**Table 2:** Characteristics of eligible individuals with and without hypernatremia as evidenced by recorded serum sodium levels.

		Total	Not hypernatremic during hospitalization	Hypernatremic during hospitalization
Variable	Category	*N* = 1 813 356	*N* = 1 680 793	N = 132 563
Age category	18–49 years	233 118 (12.9)	228 814 (13.6)	4304 (3.2)
	50–64 years	269 105 (14.8)	257 549 (15.3)	11 556 (8.7)
	65–79 years	657 411 (36.3)	618 453 (36.8)	38 958 (29.4)
	≥80 years	653 722 (36.1)	575 977 (34.3)	77 745 (58.6)
Male		953 378 (52.6)	888 911 (52.9)	64 467 (48.6)
Minimum sodium concentration on admission day (mmol/l)		139 [136–141]	139 [136–141]	142 [138–146]
Minimum sodium concentration during hospitalization (mmol/l)		138 [135–140]	138 [135–140]	138 [134–142]
Minimum sodium concentration after admission day (mmol/l)		138 [135–140]	138 [135–140]	138 [135–142]
Maximum sodium concentration on admission day (mmol/l)		139 [137–141]	139 [136–141]	142 [139–146]
Maximum sodium concentration during hospitalization (mmol/l)		141 [139–143]	141 [139–142]	148 [146–152]
Maximum sodium concentration after admission day (mmol/l)		141 [139–143]	140 [139–142]	148 [146–152]
Number of days with sodium measurement per week		2.0 [1.2–3.0]	2.0 [1.2–2.9]	2.2 [1.5–3.2]
Admission-precipitating diagnosis	Infectious disease	40 562 (2.2)	36 920 (2.2)	3642 (2.7)
based on ICD-10 code	Malignancy	335 296 (18.5)	322 988 (19.2)	12 308 (9.3)
	Blood disorders	15 052 (0.8)	13 785 (0.8)	1267 (1.0)
	Endocrinological	65 466 (3.6)	57 209 (3.4)	8257 (6.2)
	Mental disorders	7785 (0.4)	7283 (0.4)	502 (0.4)
	Neurological	43 666 (2.4)	40 689 (2.4)	2977 (2.2)
	Ophthalmological	5791 (0.3)	5702 (0.3)	89 (0.1)
	Otological	16 750 (0.9)	16 492 (1.0)	258 (0.2)
	Cardiovascular	361 762 (19.9)	324 412 (19.3)	37 350 (28.2)
	Respiratory	149 954 (8.3)	128 679 (7.7)	21 275 (16.0)
	Digestive	237 913 (13.1)	227 360 (13.5)	10 553 (8.0)
	Dermatological	18 510 (1.0)	17 227 (1.0)	1283 (1.0)
	Musculoskeletal	90 913 (5.0)	86 404 (5.1)	4509 (3.4)
	Genitourinary	121 383 (6.7)	111 446 (6.6)	9937 (7.5)
	Pregnancy	39 033 (2.2)	38 964 (2.3)	69 (0.1)
	Perinatal	50 (0.0)	50 (0.0)	0 (0.0)
	Congenital	2866 (0.2)	2730 (0.2)	136 (0.1)
	Symptoms/signs	21 432 (1.2)	19 340 (1.2)	2092 (1.6)
	Injury/poisoning	200 981 (11.1)	188 191 (11.2)	12 790 (9.6)
	New diseases	36 591 (2.0)	33 348 (2.0)	3243 (2.4)
	External causes	4 (0.0)	3 (0.0)	1 (0.0)
	Examinations	1596 (0.1)	1571 (0.1)	25 (0.0)
BMI (kg/m^2^)		22.2 [19.9–24.6]	22.2 [19.9–24.7]	21.2 [18.8–23.8]
BMI category	<18.5 kg/m^2^	191 684 (10.6)	171 131 (10.2)	20 553 (15.5)
	18.5–<25 kg/m^2^	943 232 (52.0)	883 524 (52.6)	59 708 (45.0)
	≥25 kg/m^2^	325 582 (18.0)	309 686 (18.4)	15 896 (12.0)
	Missing	352 858 (19.5)	316 452 (18.8)	36 406 (27.5)
Smoking history	Non-smoker	1 150 678 (63.5)	1 063 144 (63.3)	87 534 (66.0)
	Current/past smoker	460 159 (25.4)	435 492 (25.9)	24 667 (18.6)
	Missing	202 519 (11.2)	182 157 (10.8)	20 362 (15.4)
Consciousness level	Alert	1 548 278 (85.4)	1 463 553 (87.1)	84 725 (63.9)
	Not clear	265 078 (14.6)	217 240 (12.9)	47 838 (36.1)
Charlson comorbidity index		0 [0–2]	0 [0–2]	0 [0–2]
Activities of daily living	Independent	971 959 (53.6)	941 996 (56.0)	29 963 (22.6)
	Dependent	700 965 (38.7)	612 066 (36.4)	88 899 (67.1)
	Missing	140 432 (7.7)	126 731 (7.5)	13 701 (10.3)
Unscheduled admission		1 087 416 (60.0)	979 310 (58.3)	108 106 (81.6)
Ambulance use		502 607 (27.7)	435 602 (25.9)	67 005 (50.5)
In-hospital death		112 226 (6.2)	79 373 (4.7)	32 853 (24.8)

Data are presented *N* (%) for categorical variables and median [1st–3rd quartile] for continuous variables.

BMI, body mass index; ICD-10, the International Classification of Diseases, Tenth Revision

**Table 3:** Frequency of admissions based on the admission-precipitating diagnosis.

Type of population	Disease frequency (%)	ICD-10 code	Disease name
All admissions (*N* = 1 813 356)	3.5	I50	Heart failure
	3.4	I63	Cerebral infarction
	3.1	I20	Angina pectoris
	2.8	S72	Fracture of femur
	2.6	C34	Malignant neoplasm of bronchus and lung
	2.3	K80	Cholelithiasis
	2.2	J69	Pneumonitis due to solids and liquids
	2.0	U07	COVID-19 infection
	1.8	C18	Malignant neoplasm of colon
	1.6	C16	Malignant neoplasm of stomach
Individuals with hyponatremia (serum sodium	4.6	I50	Heart failure
<135 mmol/l) during hospitalization (*N* = 419 470)	4.4	J69	Pneumonitis due to solids and liquids
	3.2	S72	Fracture of femur
	2.8	I63	Cerebral infarction
	2.8	C34	Malignant neoplasm of bronchus and lung
	2.6	U07	COVID-19 infection
	2.4	J18	Pneumonia, organism unspecified
	2.0	N39	Other disorders of urinary system
	1.9	C18	Malignant neoplasm of colon
	1.9	J15	Bacterial pneumonia, not elsewhere classified
Individuals with hypernatremia (serum sodium	9.1	I50	Heart failure
>145 mmol/l) during hospitalization (*N* = 132 563)	6.9	J69	Pneumonitis due to solids and liquids
	4.0	I63	Cerebral infarction
	3.6	S72	Fracture of femur
	2.9	J18	Pneumonia, organism unspecified
	2.9	E86	Volume depletion
	2.8	I71	Aortic aneurysm and dissection
	2.4	U07	COVID-19 infection
	2.4	I61	Intracerebral hemorrhage
	2.4	N39	Other disorders of urinary system

ICD-10, the International Classification of Diseases, Tenth Revision; COVID-19, coronavirus disease 2019

### Validity of Diagnosis Procedure Combination data for identifying a laboratory diagnosis of hyponatremia

Table 4 shows the validity indices of the DPC data for the identification of hyponatremia. Overall, the ICD-10 code (E87.1) showed a low sensitivity and high specificity; the sensitivity was 4.1%, and the specificity was 99.9% to identify serum sodium levels <135 mmol/l during hospitalization. The LR+, LR−, and DOR were 41.1, 0.96, and 42.8, respectively. The PPV was 92.5%, and NPV was 77.6% in this research context. When moderate and severe hyponatremia were defined by serum sodium levels of <130 and <125 mmol/l, the sensitivities increased to 11.4% and 23.8%, respectively, while maintaining specificity >99% across the various cutoff points. Compared to using all diagnoses to identify hyponatremia during hospitalization, when using ICD-10-based diagnosis, the sensitivity was higher on the day of admission and lower post-admission day.

**Table 4:** Validity of DPC data for identifying hyponatremia based on various cutoff values and the timing of the registered diagnosis.

Timing	Cutoff, mmol/l	Disease frequency based on sodium data (%)	Diagnosis frequency based on DPC data (%)	Sensitivity (%)	Specificity (%)	PPV (%)	NPV (%)	LR+	LR−	DOR
During	<135	23.1	1.0	4.1	99.9	92.5	77.6	41.1	0.96	42.8
hospitalization	<130	6.9	1.0	11.4	99.8	77.9	93.8	47.2	0.89	53.1
(*N* = 1 813 356)	<125	2.1	1.0	23.8	99.5	50.4	98.3	46.3	0.77	60.4
On admission day	<135	14.9	0.9	5.4	99.9	90.1	85.8	51.9	0.95	54.8
(*N* = 1 351 760)	<130	4.2	0.9	16.4	99.8	76.9	96.5	76.3	0.84	91.0
	<125	1.4	0.9	33.9	99.6	52.1	99.1	78.1	0.66	118.0
After admission day	<135	22.2	0.3	1.3	>99.9	88.3	78.0	26.5	0.99	26.9
(*N* = 1 504 321)	<130	6.4	0.3	3.2	99.9	64.5	93.7	26.4	0.97	27.2
	<125	1.9	0.3	5.5	99.8	32.0	98.2	24.8	0.95	26.2

DPC, Diagnosis Procedure Combination; NPV, negative predictive value; PPV, positive predictive value; LR+: positive likelihood ratio; LR−: negative likelihood ratio; DOR: diagnostic odds ratio

Table 5 presents the results of stratified, supplementary, and sensitivity analyses. In comparison to the analysis of the patients aged <75 years, the analysis of those aged ≥75 years yielded a higher sensitivity, while the specificity was similarly high. The sensitivity was lowest for patients with neoplasms, followed by those with cardiovascular and respiratory diseases. When the cutoffs for hyponatremia were lowered to 120, 115, 110, and 105 mmol/l, the sensitivity increased, and the specificity decreased as the cutoff became more extreme, similar to the observations in the main analysis. The results of the sensitivity analyses were similar to those of the primary analysis.

**Table 5:** Validity of DPC data for identifying hyponatremia based on various cutoff values and the timing of the registered diagnosis, in stratified, supplementary, and sensitivity analyses.

Type of analysis, strata or conditions	Timing	Cutoff, mmol/l	Disease frequency based on sodium data (%)	Diagnosis frequency based on DPC data (%)	Sensitivity (%)	Specificity (%)	PPV (%)	NPV (%)	LR+	LR−	DOR
Aged <75 years	During hospitalization	<135	17.3	0.7	3.5	99.9	91.6	83.2	52.3	0.97	54.2
	(*N* = 906 159)	<130	4.6	0.7	11.1	99.8	76.6	95.9	68.4	0.89	76.8
		<125	1.4	0.7	24.6	99.7	51.5	98.9	75.8	0.76	100.0
	On admission day	<135	11.8	0.6	4.9	99.9	90.2	88.7	68.9	0.95	72.4
	(*N* = 625 261)	<130	3.1	0.6	16.0	99.8	76.5	97.4	103.0	0.84	122.0
		<125	1.0	0.6	33.4	99.7	54.0	99.3	112.0	0.67	168.0
	After admission day	<135	16.6	0.2	1.0	>99.9	86.4	83.5	31.9	0.99	32.2
	(*N* = 730 701)	<130	4.2	0.2	2.9	99.9	61.1	95.9	35.6	0.97	36.7
		<125	1.2	0.2	5.1	99.9	30.1	98.9	36.2	0.95	38.1
Aged ≥75 years	During hospitalization	<135	29.0	1.4	4.4	99.9	93.0	71.9	32.4	0.96	33.8
	(*N* = 907 197)	<130	9.3	1.4	11.5	99.7	78.5	91.6	35.5	0.89	40.0
		<125	2.9	1.4	23.4	99.3	49.8	97.7	33.2	0.77	43.0
	On admission day	<135	17.6	1.1	5.7	99.9	90.1	83.2	42.4	0.94	44.9
	(*N* = 726 499)	<130	5.1	1.1	16.6	99.7	77.1	95.7	62.1	0.84	74.3
		<125	1.7	1.1	34.2	99.4	51.1	98.9	61.9	0.66	93.4
	After admission day	<135	27.4	0.4	1.4	99.9	89.1	72.8	21.7	0.99	22.0
	(*N* = 773 620)	<130	8.5	0.4	3.4	99.8	65.9	91.7	20.8	0.97	21.4
		<125	2.5	0.4	5.7	99.7	32.8	97.6	19.0	0.95	20.1
Analysis stratified by	During hospitalization	<135	22.9	0.5	1.9	99.9	86.8	77.5	22.2	0.98	22.6
admission-necessitating	(N = 335 296)	<130	6.6	0.5	5.1	99.8	68.6	93.7	31.0	0.95	32.6
diagnosis, neoplasms		<125	1.8	0.5	10.8	99.7	39.6	98.4	35.4	0.90	39.6
	On admission day	<135	13.5	0.4	2.0	99.9	75.9	86.7	20.1	0.98	20.5
	(*N* = 194 310)	<130	3.5	0.4	5.4	99.8	53.5	96.7	31.5	0.95	33.2
		<125	0.9	0.4	11.5	99.7	29.5	99.2	46.0	0.89	51.8
	After admission day	<135	24.4	0.3	1.1	>99.9	90.3	75.8	29.0	0.99	29.3
	(*N* = 270 295)	<130	6.9	0.3	3.0	99.9	71.8	93.3	34.5	0.97	35.5
		<125	1.9	0.3	5.9	99.8	38.0	98.2	31.9	0.94	33.8
Analysis stratified by	During hospitalization	<135	20.7	0.6	2.5	99.9	90.7	79.7	37.4	0.98	38.3
admission-necessitating	(*N* = 361 762)	<130	5.7	0.6	6.7	99.8	66.7	94.6	33.1	0.94	35.4
diagnosis, cardiovascular		<125	1.6	0.6	12.2	99.6	33.6	98.6	31.8	0.88	36.0
	On admission day	<135	10.0	0.3	2.2	99.9	80.6	90.2	37.6	0.98	38.4
	(*N* = 300 913)	<130	2.4	0.3	6.8	99.9	61.3	97.7	63.7	0.93	68.3
		<125	0.7	0.3	13.0	99.8	35.4	99.4	74.9	0.87	86.0
	After admission day	<135	21.0	0.4	1.7	>99.9	90.5	79.2	35.9	0.98	36.5
	(*N* = 289 463)	<130	5.7	0.4	4.1	99.8	59.2	94.5	24.1	0.96	25.1
		<125	1.4	0.4	6.4	99.7	23.6	98.6	21.1	0.94	22.5
Analysis stratified by	During hospitalization	<135	35.5	1.6	4.3	99.8	93.4	65.4	25.8	0.96	27.0
admission-necessitating	(*N* = 149 954)	<130	12.5	1.6	10.3	99.6	78.8	88.6	26.0	0.90	28.8
diagnosis, respiratory		<125	4.2	1.6	18.0	99.1	45.7	96.5	19.4	0.83	23.5
	On admission day	<135	24.4	1.2	4.3	99.9	90.9	76.4	30.9	0.96	32.2
	(*N* = 134 818)	<130	7.4	1.2	11.7	99.7	75.6	93.4	38.5	0.89	43.5
		<125	2.3	1.2	21.6	99.3	43.6	98.1	32.3	0.79	40.9
	After admission day	<135	29.5	0.6	1.6	99.9	84.7	70.8	13.2	0.99	13.4
	(*N* = 135 778)	<130	10.0	0.6	3.6	99.8	63.4	90.3	15.6	0.97	16.1
		<125	3.1	0.6	6.0	99.6	32.9	97.0	15.2	0.94	16.1
Analysis stratified by	During hospitalization	<135	22.2	1.3	5.3	99.9	93.4	78.7	49.8	0.95	52.5
admission-necessitating	(*N* = 966 344)	<130	6.7	1.3	15.3	99.7	80.8	94.3	59.0	0.85	69.5
diagnosis, other		<125	2.2	1.3	32.5	99.4	55.6	98.5	56.6	0.68	83.4
	On admission day	<135	15.6	1.3	7.4	99.9	91.9	85.4	61.4	0.93	66.3
	(*N* = 721 719)	<130	4.5	1.3	22.4	99.7	80.3	96.5	86.8	0.78	111.0
		<125	1.6	1.3	44.7	99.4	56.7	99.1	81.3	0.56	146.0
	After admission day	<135	20.6	0.3	1.1	>99.9	87.7	79.6	27.4	0.99	27.7
	(*N* = 808 785)	<130	6.0	0.3	2.9	99.9	65.0	94.2	29.3	0.97	30.1
		<125	1.8	0.3	5.0	99.8	33.8	98.3	28.1	0.95	29.5
Supplementary analysis,	During hospitalization	<120	0.67	1.0	40.1	99.3	26.6	99.6	53.5	0.60	88.7
more extreme cutoffs	(*N* = 1 813 356)	<115	0.21	1.0	50.3	99.1	10.5	99.9	55.3	0.50	110.0
		<110	0.06	1.0	57.3	99.0	3.7	>99.9	58.7	0.43	136.0
		<105	0.02	1.0	59.2	99.0	0.9	>99.9	58.9	0.41	143.0
	On admission day	<120	0.49	0.9	53.2	99.4	29.0	99.8	83.5	0.47	177.0
	(*N* = 1 351 760)	<115	0.17	0.9	62.0	99.2	12.1	99.9	78.8	0.38	206.0
		<110	0.06	0.9	69.4	99.1	4.5	>99.9	81.3	0.31	263.0
		<105	0.01	0.9	68.9	99.1	1.1	>99.9	78.0	0.31	248.0
	After admission day	<120	0.52	0.3	8.0	99.7	12.9	99.5	28.3	0.92	30.7
	(*N* = 1 504 321)	<115	0.14	0.3	8.0	99.7	3.5	99.9	25.7	0.92	27.8
		<110	0.04	0.3	8.5	99.7	1.0	>99.9	26.7	0.92	29.1
		<105	0.01	0.3	9.7	99.7	0.2	>99.9	30.2	0.91	33.3
Sensitivity analysis 1,	During hospitalization	<135	21.2	1.0	4.3	99.9	93.0	79.5	49.6	0.96	51.8
first-time admissions	(*N* = 1 156 291)	<130	6.2	1.0	12.4	99.8	78.4	94.5	55.0	0.88	62.7
		<125	2.0	1.0	25.9	99.5	51.6	98.5	53.5	0.75	71.8
	On admission day	<135	13.7	0.9	5.8	99.9	91.0	86.9	63.6	0.94	67.4
	(*N* = 848 826)	<130	3.8	0.9	18.2	99.8	78.4	96.9	93.0	0.82	114.0
		<125	1.3	0.9	37.1	99.6	54.3	99.2	91.8	0.63	145.0
	After admission day	<135	20.0	0.3	1.4	>99.9	88.9	80.3	32.2	0.99	32.6
	(*N* = 964 571)	<130	5.7	0.3	3.5	99.9	64.2	94.5	29.8	0.97	30.9
		<125	1.7	0.3	5.9	99.8	31.6	98.4	27.3	0.94	28.9
Sensitivity analysis 2,	During hospitalization	<135	21.5	1.0	4.3	99.9	92.2	79.2	43.3	0.96	45.2
excluding those with	(*N* = 1 431 877)	<130	6.5	1.0	12.0	99.8	78.0	94.2	51.2	0.88	58.0
diabetes,		<125	2.1	1.0	24.7	99.5	50.9	98.4	49.4	0.76	65.3
hypertriglyceridemia, or	On admission day	<135	13.8	0.9	5.8	99.9	89.7	86.9	54.7	0.94	58.1
multiple myeloma	(*N* = 1 068 544)	<130	3.9	0.9	17.5	99.8	77.0	96.7	82.4	0.83	99.7
		<125	1.3	0.9	35.5	99.6	52.4	99.1	82.6	0.65	128.0
	After admission day	<135	20.8	0.3	1.3	>99.9	88.0	79.4	27.9	0.99	28.3
	(*N* = 1 173 835)	<130	6.1	0.3	3.3	99.9	64.7	94.1	28.0	0.97	28.9
		<125	1.8	0.3	5.6	99.8	32.9	98.3	26.5	0.95	28.0

DPC, Diagnosis Procedure Combination; NPV, negative predictive value; PPV, positive predictive value; LR+: positive likelihood ratio; LR−: negative likelihood ratio; DOR: diagnostic odds ratio

Note: For results on the disease frequency based on sodium data in the supplementary analysis, prevalence is shown with more significant figures for accuracy.

### Validity of Diagnosis Procedure Combination data for identifying a laboratory diagnosis of hypernatremia

Table 6 presents the validity of the DPC data for hypernatremia. Overall, the ICD-10 code (E87.2) exhibited low sensitivity and high specificity. Identifying serum sodium levels of >145 mmol/l during hospitalization yielded a sensitivity of 2.2% and specificity of >99.9%; the LR+, LR−, and DOR were 350, 0.98, and 358, respectively; and the PPV and NPV in this research context were reported at 96.5% and 92.8%, respectively. When serum sodium levels >150 and >155 mmol/l were used to define moderate and severe hypernatremia, the sensitivities increased to 5.9% and 9.3%, respectively, while maintaining specificity at 99.9%. Compared with using all diagnoses during hospitalization to identify hypernatremia, the sensitivity was higher on the admission day and lower post-admission when using an ICD-10-based diagnosis.

**Table 6:** Validity of DPC data for identifying hypernatremia based on various cutoff values and the timing of the registered diagnosis.

Timing	Cutoff, mmol/L	Disease frequency based on sodium data (%)	Diagnosis frequency based on DPC data (%)	Sensitivity (%)	Specificity (%)	PPV (%)	NPV (%)	LR+	LR−	DOR
During	>145	7.3	0.2	2.2	>99.9	96.5	92.8	350	0.98	358
hospitalization	>150	2.4	0.2	5.9	>99.9	86.4	97.7	259	0.94	275
(*N* = 1 813 356)	>155	1.2	0.2	9.3	99.9	68.7	98.9	181	0.91	200
On admission day	>145	2.8	0.1	4.3	>99.9	91.5	97.4	378	0.96	395
(*N* = 1 351 760)	>150	0.7	0.1	14.3	>99.9	79.8	99.4	536	0.86	625
	>155	0.4	0.1	21.7	99.9	61.8	99.7	432	0.78	552
After admission day	>145	7.6	0.1	0.8	>99.9	96.5	92.4	330	0.99	333
(*N* = 1 504 321)	>150	2.7	0.1	2.1	>99.9	83.7	97.4	187	0.98	191
	>155	1.3	0.1	3.1	>99.9	62.4	98.7	124	0.97	128

DPC, Diagnosis Procedure Combination; NPV, negative predictive value; PPV, positive predictive value; LR+: positive likelihood ratio; LR−: negative likelihood ratio; DOR: diagnostic odds ratio

The results of stratified and sensitivity analyses are shown in Table 7. Compared with the analysis of younger patients, the analysis of older ones resulted in a generally higher sensitivity, while the specificity remained similarly high. Sensitivity was lowest for patients with neoplasms, followed by those with cardiovascular and respiratory diseases. The results of the sensitivity analyses were similar to those of the primary analysis.

**Table 7:** Validity of DPC data for identifying hypernatremia based on various cutoff values and the timing of the registered diagnosis, in stratified and sensitivity analyses.

Type of analysis, strata, or conditions	Timing	Cutoff, mmol/L	Disease frequency based on sodium data (%)	Diagnosis frequency based on DPC data (%)	Sensitivity (%)	Specificity (%)	PPV (%)	NPV (%)	LR+	LR−	DOR
Aged <75 years	During hospitalization	>145	4.1	0.1	1.4	>99.9	94.5	95.9	396	0.99	402
	(*N* = 906 159)	>150	1.0	0.1	5.1	>99.9	81.8	99.1	448	0.95	472
		>155	0.5	0.1	8.6	>99.9	64.8	99.6	391	0.92	428
	On admission day	>145	1.7	0.0	2.6	>99.9	88.6	98.4	454	0.97	466
	(*N* = 625 261)	>150	0.3	0.0	13.2	>99.9	73.3	99.8	1005	0.87	1158
		>155	0.1	0.0	22.4	>99.9	55.7	99.9	1028	0.78	1324
	After admission day	>145	4.2	0.0	0.7	>99.9	94.8	95.8	416	0.99	418
	(*N* = 730 701)	>150	1.1	0.0	2.1	>99.9	82.9	98.9	424	0.98	433
		>155	0.5	0.0	3.5	>99.9	64.5	99.5	336	0.97	348
Aged ≥75 years	During hospitalization	>145	10.5	0.3	2.4	>99.9	97.0	89.7	275	0.98	281
	(*N* = 907 197)	>150	3.8	0.3	6.1	>99.9	87.5	96.4	177	0.94	188
		>155	1.9	0.3	9.5	99.9	69.7	98.2	117	0.91	129
	On admission day	>145	3.7	0.2	5.0	>99.9	92.1	96.5	303	0.95	319
	(*N* = 726 499)	>150	1.1	0.2	14.5	>99.9	81.1	99.0	378	0.86	442
		>155	0.6	0.2	21.6	99.9	63.0	99.5	288	0.79	368
	After admission day	>145	10.8	0.1	0.9	>99.9	96.9	89.2	258	0.99	260
	(*N* = 773 620)	>150	4.1	0.1	2.0	>99.9	83.9	96.0	121	0.98	124
		>155	2.1	0.1	3.0	>99.9	61.9	98.0	77	0.97	79
Analysis stratified by	During hospitalization	>145	3.7	0.0	0.7	>99.9	92.5	96.4	322	0.99	325
admission-necessitating	(*N* = 335 296)	>150	0.9	0.0	2.5	>99.9	81.7	99.1	483	0.98	495
diagnosis, neoplasms		>155	0.4	0.0	3.6	>99.9	57.0	99.6	300	0.96	311
	On admission day	>145	1.7	0.0	0.6	>99.9	71.4	98.3	147	0.99	148
	(*N* = 194 310)	>150	0.2	0.0	3.2	>99.9	50.0	99.8	445	0.97	459
		>155	0.1	0.0	2.3	>99.9	14.3	99.9	188	0.98	193
	After admission day	>145	3.7	0.0	0.6	>99.9	98.2	96.4	1445	0.99	1453
	(*N* = 270 295)	>150	1.0	0.0	1.8	>99.9	89.3	99.0	792	0.98	806
		>155	0.5	0.0	2.7	>99.9	66.1	99.5	382	0.97	392
Analysis stratified by	During hospitalization	>145	10.3	0.1	0.9	>99.9	96.4	89.8	230	0.99	232
admission-necessitating	(*N* = 361 762)	>150	3.3	0.1	2.5	>99.9	81.5	96.8	130	0.98	134
diagnosis, cardiovascular		>155	1.6	0.1	3.8	>99.9	60.2	98.5	96	0.96	100
	On admission day	>145	3.0	0.0	0.8	>99.9	70.1	97.0	76	0.99	77
	(*N* = 300 913)	>150	0.5	0.0	3.7	>99.9	51.4	99.5	213	0.96	221
		>155	0.2	0.0	6.4	>99.9	34.6	99.8	274	0.94	292
	After admission day	>145	11.1	0.1	0.7	>99.9	97.1	88.9	271	0.99	273
	(*N* = 289 463)	>150	3.8	0.1	1.9	>99.9	84.1	96.3	134	0.98	136
		>155	1.8	0.1	2.9	>99.9	61.6	98.2	87	0.97	89
Analysis stratified by	During hospitalization	>145	14.2	0.4	2.5	>99.9	97.1	86.1	204	0.98	209
admission-necessitating	(*N* = 149 954)	>150	6.0	0.4	5.3	99.9	86.2	94.3	98	0.95	103
diagnosis, respiratory		>155	3.0	0.4	8.2	99.9	67.4	97.2	66	0.92	72
	On admission day	>145	4.4	0.2	5.1	>99.9	92.5	95.9	272	0.95	286
	(*N* = 134 818)	>150	1.7	0.2	11.4	>99.9	80.7	98.5	244	0.89	275
		>155	0.9	0.2	17.0	99.9	65.1	99.2	202	0.83	243
	After admission day	>145	14.3	0.2	1.1	>99.9	97.6	85.8	244	0.99	247
	(*N* = 135 778)	>150	6.1	0.2	2.0	>99.9	81.3	94.0	67	0.98	68
		>155	3.1	0.2	3.0	99.9	59.3	97.0	46	0.97	48
Analysis stratified by	During hospitalization	>145	6.4	0.2	3.1	>99.9	96.6	93.8	411	0.97	424
admission-necessitating	(*N *= 966 344)	>150	2.0	0.2	8.7	>99.9	87.7	98.1	341	0.91	374
diagnosis, other		>155	1.0	0.2	13.7	99.9	71.2	99.1	234	0.86	271
	On admission day	>145	2.7	0.2	6.4	>99.9	93.4	97.5	516	0.94	551
	(*N* = 721 719)	>150	0.8	0.2	19.1	>99.9	82.5	99.4	594	0.81	733
		>155	0.4	0.2	27.7	99.9	64.2	99.7	422	0.72	583
	After admission day	>145	6.5	0.1	0.9	>99.9	95.4	93.5	296	0.99	299
	(*N* = 808 785)	>150	2.2	0.1	2.2	>99.9	83.9	97.8	228	0.98	233
		>155	1.1	0.1	3.3	>99.9	63.8	98.9	154	0.97	159
Sensitivity analysis 1,	During hospitalization	>145	7.3	0.2	2.3	>99.9	96.4	92.8	336	0.98	343
first-time admissions	(*N* = 1 156 291)	>150	2.5	0.2	6.1	>99.9	86.2	97.7	246	0.94	262
		>155	1.2	0.2	9.7	99.9	68.7	98.9	176	0.90	195
	On admission day	>145	2.8	0.1	4.6	>99.9	92.3	97.3	412	0.95	432
	(*N* = 848 826)	>150	0.8	0.1	14.8	>99.9	79.8	99.4	519	0.85	608
		>155	0.4	0.1	22.5	99.9	61.7	99.7	419	0.78	540
	After admission day	>145	7.6	0.1	0.9	>99.9	96.4	92.4	324	0.99	327
	(*N* = 964 571)	>150	2.7	0.1	2.2	>99.9	83.7	97.3	183	0.98	187
		>155	1.4	0.1	3.3	>99.9	62.9	98.7	123	0.97	128
Sensitivity analysis 2,	During hospitalization	>145	7.2	0.2	2.0	>99.9	96.2	92.9	325	0.98	332
excluding those with	(*N* = 1 431 877)	>150	2.3	0.2	5.6	>99.9	84.7	97.8	236	0.94	250
diabetes,		>155	1.1	0.2	9.1	99.9	66.4	99.0	175	0.91	193
hypertriglyceridemia, or	On admission day	>145	2.8	0.1	4.1	>99.9	91.4	97.3	361	0.96	377
multiple myeloma	(*N* = 1 068 544)	>150	0.7	0.1	14.0	>99.9	78.9	99.4	520	0.86	605
		>155	0.4	0.1	21.8	>99.9	60.9	99.7	438	0.78	560
	After admission day	>145	7.5	0.1	0.7	>99.9	95.7	92.6	276	0.99	278
	(*N* = 1 173 835)	>150	2.6	0.1	1.8	>99.9	80.9	97.5	162	0.98	165
		>155	1.2	0.1	2.7	>99.9	58.1	98.8	111	0.97	114

DPC, Diagnosis Procedure Combination; NPV, negative predictive value; PPV, positive predictive value; LR+: positive likelihood ratio; LR−: negative likelihood ratio; DOR: diagnostic odds ratio

### Identification of hyponatremia and hypernatremia when sodium levels are corrected for glucose, triglyceride, and total protein levels

**Figure 3:**
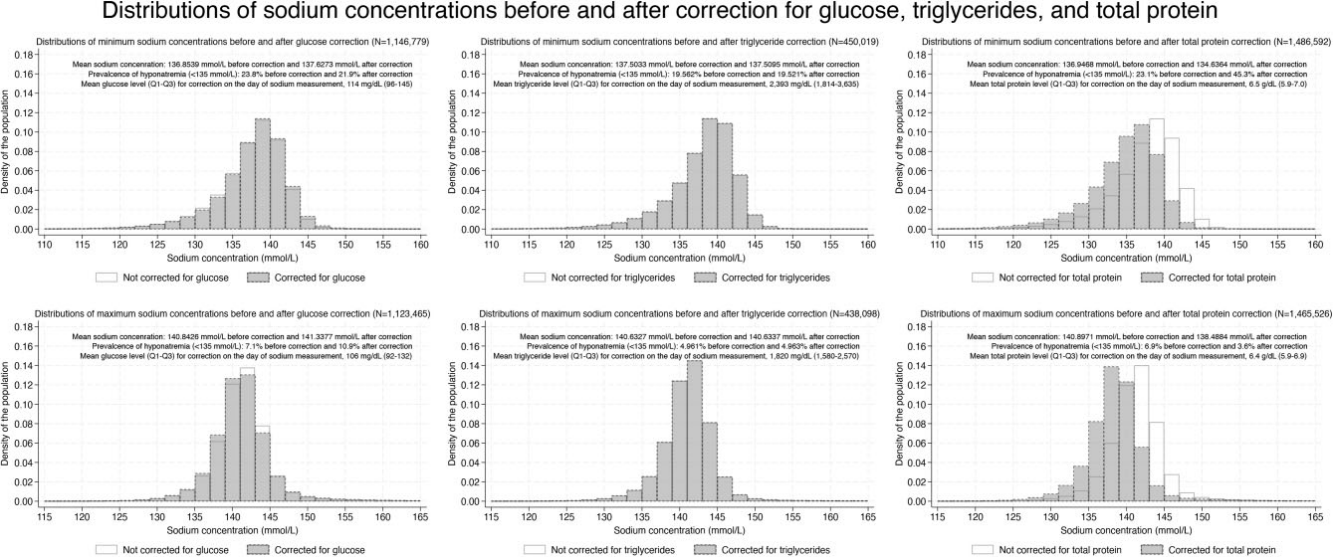
Histograms showing sodium concentrations for minimum (upper panels) and maximum values (lower panels) with and without corrections for glucose (left panels), triglycerides (center panels), and total protein (right panels). In each panel, mean sodium levels, the prevalence of hyponatremia (sodium <135 mmol/l, upper panels) or hypernatremia (sodium >145 mmol/l, lower panels) before and after corrections, and mean concentrations of glucose, triglycerides, and total protein used for corrections are described in right upper areas. Note that for the triglyceride correction, due to a smaller sample size, prevalence is shown with more significant figures for accuracy.

Figure 3 and Tables 8 and 9 present results with and without corrections for glucose, triglyceride, and total protein levels among individuals with available data for each parameter. Both when sodium levels were lowest and highest, mean sodium levels became higher with corrections for glucose or triglycerides, while the corresponding values became lower with corrections for total protein (Table 8). Although the sensitivity and specificity remained largely unchanged after the corrections (Table 10), the prevalence of hyponatremia or hypernatremia was affected by the corrections, and the extent of this effect varied among the types of correction.

**Table 8:** Mean sodium levels when sodium levels were lowest or highest during hospitalization before and after corrections for glucose, triglyceride, and total protein levels.

Data on sodium levels when sodium levels were lowest during hospitalization		
Individuals with available glucose levels when sodium levels were lowest during hospitalization (*N* = 1 146 779)		
	Mean sodium level (mmol/l)	95% confidence interval
Sodium levels without glucose correction	136.8539	136.8451–136.8628
Sodium levels with glucose correction	137.6273	137.6184–137.6361
Individuals with available triglyceride levels when sodium levels were lowest during hospitalization (*N* = 450 019)		
	Mean sodium level (mmol/l)	95% confidence interval
Sodium levels without triglyceride correction	137.5033	137.4894–137.5172
Sodium levels with triglyceride correction	137.5095	137.4957–137.5234
Individuals with available total protein levels when sodium levels were lowest during hospitalization (*N* = 1 486 592)		
	Mean sodium level (mmol/l)	95% confidence interval
Sodium levels without total protein correction	136.9468	136.9392–136.9543
Sodium levels with total protein correction	134.6364	134.6287–134.6441
		
Data on sodium levels when sodium levels were highest during hospitalization		
		
Individuals with available glucose levels when sodium levels were highest during hospitalization (*N* = 1 123 465)		
	Mean sodium level (mmol/l)	95% confidence interval
Sodium levels without glucose correction	140.8426	140.8344–140.8507
Sodium levels with glucose correction	141.3377	141.3292–141.3463
Individuals with available triglyceride levels when sodium levels were highest during hospitalization (*N* = 450 019)		
	Mean sodium level (mmol/l)	95% confidence interval
Sodium levels without triglyceride correction	140.6327	140.6211–140.6444
Sodium levels with triglyceride correction	140.6337	140.6221–140.6454
Individuals with available total protein levels when sodium levels were highest during hospitalization (*N* = 1 465 526)		
	Mean sodium level (mmol/l)	95% confidence interval
Sodium levels without total protein correction	140.8971	140.8902–140.9041
Sodium levels with total protein correction	138.4884	138.4817–138.4952

**Table 9:** Cross-tabulations of hyponatremia and hypernatremia distributions before and after corrections for glucose, triglyceride, and total protein levels.

Hyponatremia			
Individuals with data on glucose levels when their sodium levels during hospitalization were at a minimum (*N* = 1 146 779)			
	Hyponatremia with glucose correction		
Hyponatremia without glucose correction	Absent	Present	Total
Absent	853 827	19 612	873 439
Present	41 546	231 794	273 340
Total	895 373	251 406	1 146 779
Individuals with data on triglyceride levels when their sodium levels during hospitalization were at a minimum (*N* = 450 019)			
	Hyponatremia with triglyceride correction		
Hyponatremia without triglyceride correction	Absent	Present	Total
Absent	361 988	0	361 988
Present	184	87 847	88 031
Total	362 172	87 847	450 019
Individuals with data on total protein levels when their sodium levels during hospitalization were at a minimum (*N* = 1 486 592)			
	Hyponatremia with total protein correction		
Hyponatremia without total protein correction	Absent	Present	Total
Absent	813 137	330 729	1 143 866
Present	111	342 615	342 726
Total	813 248	673 344	1 486 592
Hypernatremia			
Individuals with data on glucose levels when their sodium levels during hospitalization were at a maximum (*N* = 1 123 465)			
	Hypernatremia with glucose correction		
Hypernatremia without glucose correction	Absent	Present	Total
Absent	1 000 945	42 768	1 043 713
Present	510	79 242	79 752
Total	1 001 455	122 010	1 123 465
Individuals with data on triglyceride levels when their sodium levels during hospitalization were at a maximum (*N* = 438 098)			
	Hypernatremia with triglyceride correction		
Hypernatremia without triglyceride correction	Absent	Present	Total
Absent	416 354	10	416 364
Present	0	21 734	21 734
Total	416 354	21 744	438 098
Individuals with data on total protein levels when their sodium levels during hospitalization were at a maximum (*N* = 1 465 526)			
	Hypernatremia with total protein correction		
Hypernatremia without total protein correction	Absent	Present	Total
Absent	1 364 021	79	1 364 100
Present	49 383	52 043	101 426
Total	1 413 404	52 122	1 465 526

**Table 10:** Validity of DPC data for identifying hyponatremia and hypernatremia based on various cutoff values and corrections for glucose, triglycerides, or total protein in supplementary analyses.

Hyponatremia											
Type of population	Presence of correction	Cutoff, mmol/l	Disease frequency (%)	Diagnosis frequency (%)	Sensitivity (%)	Specificity (%)	PPV (%)	NPV (%)	LR+	LR−	DOR
Available glucose levels (*N* = 1 146 779)	Not corrected for glucose	<135	23.8	1.2	4.5	99.9	92.4	77.0	39.1	0.96	40.9
		<130	7.3	1.2	12.5	99.7	78.7	93.5	46.8	0.88	53.3
		<125	2.3	1.2	25.9	99.4	51.8	98.3	45.1	0.75	60.5
	Corrected for glucose (correction performed in 1 146 779)	<135	21.9	1.2	4.8	99.9	90.5	78.9	33.9	0.95	35.6
		<130	6.6	1.2	13.3	99.7	75.4	94.2	43.4	0.87	49.9
		<125	2.1	1.2	27.1	99.4	49.8	98.4	45.4	0.73	61.9
Available triglyceride levels (*N* = 450 019)	Not corrected for high triglycerides	<135	19.6	1.1	5.4	99.9	94.4	81.3	68.8	0.95	72.6
		<130	6.0	1.1	15.3	99.8	82.4	94.8	72.6	0.85	85.5
		<125	2.1	1.1	31.1	99.5	57.1	98.6	63.4	0.69	91.5
	Corrected for high triglycerides (correction performed in 361)	<135	19.5	1.1	5.4	99.9	94.2	81.3	67.4	0.95	71.2
		<130	6.0	1.1	15.3	99.8	82.2	94.8	72.2	0.85	85.1
		<125	2.0	1.1	31.2	99.5	57.0	98.6	63.5	0.69	91.8
Available total protein levels (*N* = 1 486 592)	Not corrected for total protein	<135	23.1	1.0	4.1	99.9	92.6	77.7	42.0	0.96	43.8
		<130	6.9	1.0	11.6	99.8	78.4	93.8	49.0	0.89	55.2
		<125	2.1	1.0	24.4	99.5	51.0	98.4	47.8	0.76	62.9
	Corrected for total protein (correction performed in 1 486 592)	<135	45.3	1.0	2.2	99.9	96.4	55.2	32.5	0.98	33.2
		<130	14.0	1.0	6.4	99.9	87.5	86.8	43.1	0.94	45.9
		<125	4.3	1.0	15.6	99.6	65.4	96.4	42.4	0.85	50.1
Hypernatremia											
Type of population	Presence of correction	Cutoff, mmol/L	Disease frequency (%)	Diagnosis frequency (%)	Sensitivity (%)	Specificity (%)	PPV (%)	NPV (%)	LR+	LR−	DOR
Available glucose levels (*N* = 1 123 465)	Not corrected for glucose	>145	7.1	0.2	2.6	>99.9	96.9	93.1	410	0.98	421
		>150	2.4	0.2	6.8	>99.9	87.7	97.7	289	0.93	310
		>155	1.2	0.2	10.7	99.9	69.7	98.9	187	0.89	209
	Corrected for glucose (correction performed in 1 123 465)	>145	10.9	0.2	1.7	>99.9	97.3	89.3	300	0.98	305
		>150	3.2	0.2	5.4	>99.9	90.6	97.0	295	0.95	311
		>155	1.6	0.2	8.8	>99.9	75.7	98.5	191	0.91	210
Available triglyceride levels (*N* = 438 098)	Not corrected for high triglycerides	>145	5.0	0.1	2.8	>99.9	96.5	95.2	523	0.97	538
		>150	1.3	0.1	9.2	>99.9	86.7	98.8	480	0.91	529
		>155	0.7	0.1	14.2	>99.9	70.5	99.4	337	0.86	392
	Corrected for high triglycerides (correction performed in 71)	>145	5.0	0.1	2.8	>99.9	96.5	95.2	523	0.97	538
		>150	1.3	0.1	9.2	>99.9	86.7	98.8	480	0.91	529
		>155	0.7	0.1	14.2	>99.9	70.5	99.4	336	0.86	392
Available total protein levels (*N* = 1 465 526)	Not corrected for total protein	>145	6.9	0.2	2.2	>99.9	96.7	93.2	390	0.98	399
		>150	2.2	0.2	6.1	>99.9	86.7	97.9	285	0.94	304
		>155	1.1	0.2	9.7	99.9	68.6	99.0	194	0.90	214
	Corrected for total protein (correction performed in 1 465 526)	>145	3.6	0.2	4.1	>99.9	93.5	96.6	390	0.96	407
		>150	1.5	0.2	8.0	>99.9	78.6	98.6	235	0.92	256
		>155	0.8	0.2	12.2	99.9	59.2	99.3	188	0.88	214

DPC, Diagnosis Procedure Combination; NPV, negative predictive value; PPV, positive predictive value; LR+: positive likelihood ratio; LR−: negative likelihood ratio; DOR: diagnostic odds ratio

Glucose correction decreased the prevalence of hyponatremia, while total protein correction increased that of hyponatremia (Fig. 3). Among individuals with data for glucose (*N* = 1 146 779), triglycerides (*N* = 450 019), and total protein (*N* = 1 486 592) when the sodium levels were lowest during hospitalization, 41 546 (15.2%) of 273 340, 184 (0.2%) of 88 031, 111 (0.03%) of 342 726 patients with hyponatremia without each correction were diagnosed as not having hyponatremia with correction (corrected sodium ≥135 mmol/l), respectively. The opposite occurred with these corrections. Among the individuals with available values for glucose and total protein when the sodium levels were lowest during hospitalization, 19 612 patients (2.2%) of 873 439 and 330 729 patients (28.9%) of 1 143 866 without hyponatremia without each correction were diagnosed as having hyponatremia with correction (corrected sodium <135 mmol/l), respectively. Overall, while the correction for glucose or triglyceride levels in the prevalence of hyponatremia resulted in a net decrease (from 23.8% to 21.9% and from 19.562% to 19.521%, respectively) that for total protein levels resulted in a net increase (from 23.1% to 45.3%). Conversely, while the correction for glucose or triglyceride levels, when the sodium levels were highest during hospitalization, led to a net increase in the prevalence of hypernatremia (from 7.1% to 10.9% and from 4.961% to 4.963%, respectively) that for total protein levels led to a net decrease (from 6.9% to 3.6%). The prevalence of hyponatremia or hypernatremia was not markedly affected after performing sodium correction for high triglycerides in 361 and 71 individuals in the analyses of hyponatremia and hypernatremia, respectively.

## DISCUSSION

In the present observational study using real-world data with laboratory values, we revealed that the ICD-10-based hypernatremia had a low sensitivity and high specificity to identify hypernatremia defined by serum sodium levels of >145 mmol/l. To the best of our knowledge, this is the first study to present such results using nearly 1 million admissions. In addition, we confirmed that corrections by blood glucose, triglycerides, and total protein affected the prevalence of hyponatremia and hypernatremia. By contrast, they did not largely affect the validity of both sodium abnormalities.

The validity of the ICD-10 codes for hyponatremia has been reported in several previous articles, and our analytical results agree with their findings [9, 38]. Lowering the threshold sodium value to include cases with only severe hyponatremia resulted in an increased sensitivity with a minimal impact on specificity. This finding was also consistent with those of previous studies [9, 38]. We also analyzed hyponatremia cutoffs at 110 and 105 mmol/l, ranges not covered in previous studies but important because they pose an increased risk for osmotic demyelination syndrome [37]. Furthermore, our finding of a higher sensitivity of the ICD-10 code among older patients was also observed in one previous study [10]. Notably, the present study found that the ICD-10-based diagnosis after admission had a lower sensitivity than the diagnosis present at admission. This trend was consistent with what was reported in another study [24]. The low sensitivity of the hyponatremia codes suggests that the ICD-10-based hyponatremia diagnosis should not be used for the calculation of prevalence, incidence, and absolute risk difference. While ruling in patients using ICD-10-based hyponatremia may be possible due to high LR+, ruling out patients using the same metric would be virtually impossible as the LR− value was almost one [39]. However, the high specificity and PPV indicate that the calculation of a relative risk could be acceptable as long as the disease is recorded in different groups at the same rate [40, 41]. As an example for the underestimation of hyponatremia prevalence, a previous study using the ICD-10 code reported a lower prevalence of hyponatremia (2%–6%) [5] compared to our study (23%) and another study (14%) [10], both of which used real sodium values. This study may have underreported the incidence of hyponatremia due to the use of the ICD-10 code [5]. However, the seasonality of hyponatremia, described in terms of odds ratios [5], remains interpretable because these ratios are relative indices instead of absolute ones.

Our analysis of the ICD-10 codes for hypernatremia in this study showed that, similar to hyponatremia, the registered disease code had a low sensitivity and a high specificity. We also observed a higher sensitivity for identifying more severe hypernatremia. This suggests that clinicians may have registered hypernatremia only when the sodium levels are particularly extreme. Similar to hyponatremia, the ICD-10-based diagnostic record of hypernatremia in the DPC data should not be used to calculate prevalence, incidence, or absolute risk difference due to its low sensitivity. Similar to ICD-10-based hyponatremia diagnosis, ruling in patients using ICD-10-based hypernatremia may be possible, whereas ruling out patients using the same metric would be impossible. However, the high specificity and PPV indicate that it may be acceptable to use the recorded diagnosis to calculate a relative risk in circumstances where the disease names are registered with the same probability in different patient groups. The validity of hypernatremia recorded in administrative data has not been evaluated in previous literature. Thus, further studies are warranted to confirm our findings in other countries and databases.

Evidence on hypernatremia is limited. Before databases with sodium levels were available, most articles were case reports or case series of extreme hypernatremia [27, 42, 43]. A cohort study published nearly two decades ago showed that hypernatremia was associated with older age, disturbed consciousness, lower BMI, and in-hospital mortality [44]. Recent studies utilizing data on sodium levels from 2 million patients have advanced our understanding, showing that hypernatremia is a significant determinant of hospital disposition and highlighting that extremely high sodium values predict in-hospital mortality [45, 46]. Our study contributes to this growing body of literature by demonstrating the low sensitivity of the ICD-10 diagnostic code for hypernatremia, underscoring the need for improved coding practices and the potential for enhanced patient outcomes through better identification and management of hypernatremia. Our study also uncovered that hypernatremia, like hyponatremia, was associated with increased in-hospital mortality and unscheduled hospitalization. Specifically, the coexistence of hyponatremia was associated with a 3.5-fold risk of in-hospital death, while hypernatremia was associated with a 5.3-fold risk of in-hospital death, indicating that the association for mortality may be stronger with hypernatremia than with hyponatremia. In the future, databases with available electrolyte levels, as in these studies and ours, will reveal other aspects of clinical importance of hypernatremia.

While corrections for glucose, triglyceride, and total protein levels affected the prevalence of hyponatremia and hypernatremia, they did not greatly affect the diagnostic ability of ICD-10 codes. Because the correction for glucose and triglycerides resulted in a mean increase in sodium levels and that for total protein resulted in a mean decrease in sodium levels (Table 8) regardless of highest or lowest sodium levels, correction for glucose and triglycerides led to a decrease in hyponatremia prevalence and an increase in hypernatremia prevalence, while the opposite was the case for sodium correction for total protein. The correction for total protein led to a decrease in mean sodium levels overall. This was because total protein levels in at least three-quarters of the study population (based on the 75th percentiles among patients as shown in Fig. 3) were below 7.1 g/dl, where the corrected sodium level became lower than the measured level according to the correction equation for total protein. We observed that glucose correction decreased the prevalence of hyponatremia and increased that of hypernatremia, consistent with findings from a previous study [18]. Moreover, we found that total protein correction increased the prevalence of hyponatremia by 22.2% (from 23.1% to 45.3%), which is consistent with a 27% increase among patients admitted to the critical care unit and a 36% increase among patients receiving parenteral nutrition [17]. The differences in prevalence following correction for total protein in those studies were likely due to the lower total protein levels of their included patients (ranging between 5.1–5.3 g/dl) compared to those in our study (median, 6.5 g/dl). Correction for high triglycerides did not affect the prevalence of hyponatremia and hypernatremia, probably because the correction was performed in only 361 and 71 individuals, respectively. Our study is novel in that we corrected for each value among overall hospitalized patients (i.e. not limited to patients admitted to intensive care unit), highlighting that when physicians assess the presence of hyponatremia or hypernatremia, they can easily misclassify patients if they do not consider glucose or total protein levels. This potential for misclassification can lead to underreporting of both conditions in clinical practice.

This study had a few key limitations that are worth noting. First, it was performed on data from inpatient clinical settings. The insights obtained may not be generalizable to outpatient settings. Second, we did not consider etiologies or clinical circumstances (e.g. surgeries, treatments, or the administration of fluids) that may have induced hyponatremia or hypernatremia. These types of intervention may affect disease prevalence and diagnosis frequency. In addition, we do not have access to data on sodium levels measured during blood gas testing, which usually uses direct measurement without diluting samples [47]. In this method, we can measure the osmolality gap [11], and the measured sodium concentrations are less likely to be affected by solutes [12]. Therefore, solute-corrected concentrations may not have been as accurate as concentrations measured using direct ion-specific electrodes. At the same time, it should require attention in interpreting the results of this study, in which data were used without confirming the sodium levels with direct ion-specific electrode measurements. This is because we had no access to the methods of sodium measurement used in this study. Finally, we used data from DPC hospitals that submitted laboratory and claims data to JMDC Inc. These hospitals may not represent all DPC hospitals in Japan.

This retrospective validation study revealed that ICD-10-based diagnoses of both hyponatremia and hypernatremia had low sensitivity and high specificity, suggesting that recorded hyponatremia and hypernatremia should not be used for calculations of prevalence or incidence but may be used for relative risk calculations. In addition, our analyses also suggest that the failure to adjust for blood glucose, triglycerides, or total protein may underestimate or overestimate the prevalence of both sodium disorders.

## Supplementary Material

sfae319_Supplemental_File

## Data Availability

The data underlying this article were provided by JMDC Inc. under license. Data will be shared on request to the corresponding author with permission of JMDC Inc.

## References

[1] Sato S, Yasunaga H. A review of studies using Japanese nationwide administrative claims databases. Ann Clin Epidemiol 2023;5:58–64. 10.37737/ace.23008 38505730 PMC10944998

[2] Harpe SE. Using secondary data sources for pharmacoepidemiology and outcomes research. Pharmacotherapy 2009;29:138–53. 10.1592/phco.29.2.138 19170584

[3] Terris DD, Litaker DG, Koroukian SM. Health state information derived from secondary databases is affected by multiple sources of bias. J Clin Epidemiol 2007;60:734–41. 10.1016/j.jclinepi.2006.08.012 17573990 PMC1952240

[4] Gavrielov-Yusim N, Friger M. Use of administrative medical databases in population-based research. J Epidemiol Community Health 2014;68:283–7. 10.1136/jech-2013-202744 24248997

[5] Kutz A, Ebrahimi F, Sailer CO et al. Seasonality of hypoosmolar hyponatremia in medical inpatients—data from a nationwide cohort study. J Clin Endocrinol Metab 2020;105:947–54. 10.1210/clinem/dgz320 31900477

[6] Méndez-Bailón M, Barba-Martín R, de Miguel-Yanes JM et al. Hyponatremia in hospitalised patients with heart failure in internal medicine: analysis of the Spanish national minimum basic data set (MBDS) (2005-2011). Eur J Intern Med 2015;26:603–6. 10.1016/j.ejim.2015.06.009 26118453

[7] Shea AM, Curtis LH, Szczech LA et al. Sensitivity of International Classification of Diseases codes for hyponatremia among commercially insured outpatients in the United States. BMC Nephrol 2008;9:5. 10.1186/1471-2369-9-5 18564417 PMC2447828

[8] Movig KLL, Leufkens HGM, Lenderink AW et al. Validity of hospital discharge International Classification of Diseases (ICD) codes for identifying patients with hyponatremia. J Clin Epidemiol 2003;56:530–5. 10.1016/S0895-4356(03)00006-4 12873647

[9] Gandhi S, Shariff SZ, Fleet JL et al. Validity of the international classification of diseases, 10th revision code for hospitalisation with hyponatraemia in elderly patients. BMJ Open 2012;2:e001727. 10.1136/bmjopen-2012-001727 PMC4398982 23274673

[10] Holland-Bill L, Christiansen CF, Ulrichsen SP et al. Validity of the international classification of diseases, 10th revision discharge diagnosis codes for hyponatraemia in the Danish National Registry of Patients. BMJ Open 2014;4;e004956. 10.1136/bmjopen-2014-004956 PMC4010845 24760354

[11] Aziz F, Sam R, Lew SQ et al. Pseudohyponatremia: mechanism, diagnosis, clinical associations and management. J Clin Med 2023;12:4076. 10.3390/jcm12124076 37373769 PMC10299669

[12] Liamis G, Liberopoulos E, Barkas F et al. Spurious electrolyte disorders: a diagnostic challenge for clinicians. Am J Nephrol 2013;38:50–57. 10.1159/000351804 23817179

[13] Spasovski G, Vanholder R, Allolio B et al. Clinical practice guideline on diagnosis and treatment of hyponatraemia. Eur J Endocrinol 2014;170:G1–G47. 10.1530/EJE-13-1020 24569125

[14] Steffes MW, Freier EF. A simple and precise method of determining true sodium, potassium, and chloride concentrations in hyperlipemia. J Lab Clin Med 1976;88:683–8. 965812

[15] Gómez-Hoyos E, Fernández-Peña S, Cuesta M et al. Hyponatremia in patients receiving parenteral nutrition: the importance of correcting serum sodium for total proteins. The role of the composition of parenteral nutrition in the development of hyponatremia. Eur J Clin Nutr 2018;72:446–51. 10.1038/s41430-017-0026-5 29187749

[16] Turchin A, Seifter JL, Seely EW. Clinical problem-solving. Mind the gap. N Engl J Med 2003;349:1465–9. 10.1056/NEJMcps031078 14534340

[17] Chow E, Fox N, Gama R. Effect of low serum total protein on sodium and potassium measurement by ion-selective electrodes in critically ill patients. Br J Biomed Sci 2008;65:128–31. 10.1080/09674845.2008.11732815 18986099

[18] Ng PY, Cheung RYT, Ip A et al. A retrospective cohort study on the clinical outcomes of patients admitted to intensive care units with dysnatremia. Sci Rep 2023;13:21236. 10.1038/s41598-023-48399-5 38040748 PMC10692105

[19] Nagai K, Tanaka T, Kodaira N et al. Data resource profile: JMDC claims databases sourced from medical institutions. J Gen Fam Med 2020;21:211–8. 10.1002/jgf2.367 33304714 PMC7689231

[20] Yasunaga H . Real world data in Japan: Chapter II. The Diagnosis Procedure Combination Database. Ann Clin Epidemiol 2019;1:76–79. 10.37737/ace.1.3_76

[21] Yamana H, Matsui H, Sasabuchi Y et al. Categorized diagnoses and procedure records in an administrative database improved mortality prediction. J Clin Epidemiol 2015;68:1028–35. 10.1016/j.jclinepi.2014.12.004 25596112

[22] Shigemi D, Morishima T, Yamana H et al. Validity of initial cancer diagnoses in the Diagnosis Procedure Combination data in Japan. Cancer Epidemiol 2021;74:102016. 10.1016/j.canep.2021.102016 34450452

[23] Yamana H, Moriwaki M, Horiguchi H et al. Validity of diagnoses, procedures, and laboratory data in Japanese administrative data. J Epidemiol 2017;27:476–82. 10.1016/j.je.2016.09.009 28142051 PMC5602797

[24] Yamana H, Horiguchi H, Fushimi K et al. Comparison of procedure-based and diagnosis-based identifications of severe sepsis and disseminated intravascular coagulation in administrative data. J Epidemiol 2016;26:530–7. 10.2188/jea.JE20150286 27064132 PMC5037250

[25] Yamana H, Tsuchiya A, Horiguchi H et al. Validity of a model using routinely collected data for identifying infections following gastric, colon, and liver cancer surgeries. Pharmacoepidemiol Drug Saf 2022;31:452–60. 10.1002/pds.5386 34800063

[26] Yamana H, Konishi T, Yasunaga H. Validation studies of Japanese administrative health care data: a scoping review. Pharmacoepidemiol Drug Saf 2023;32:705–17. 10.1002/pds.5636 37146098

[27] Portel L, Hilbert G, Gruson D et al. Survival with extreme hypernatremia at 209 mmol/l. Intensive Care Med 1998;24:197–8. 10.1007/PL00012683 9539084

[28] Ijaiya T, Manohar S, Lakshmi K. Therapeutic approach to the management of severe asymptomatic hyponatremia. Case Rep Nephrol 2017;2017:1371804. 28819575 10.1155/2017/1371804 PMC5551525

[29] Mahoney FI, Barthel DW. Functional evaluation: the Barthel index. Md State Med J 1965;14:61–65. 14258950

[30] Imai N, Osako K, Kaneshiro N et al. Seasonal prevalence of hyponatremia in the emergency department: impact of age. BMC Emerg Med 2018;18:41. 10.1186/s12873-018-0182-5 30442112 PMC6238288

[31] Spasovski G, Vanholder R, Allolio B et al. Clinical practice guideline on diagnosis and treatment of hyponatraemia. Nephrol Dial Transplant 2014;29:i1–i39. 10.1093/ndt/gfu040 24569496

[32] Adrogué HJ, Tucker BM, Madias NE. Diagnosis and management of hyponatremia: a review. JAMA 2022;328:280–91. 10.1001/jama.2022.11176 35852524

[33] Liamis G, Filippatos TD, Elisaf MS. Evaluation and treatment of hypernatremia: a practical guide for physicians. Postgrad Med 2016;128:299–306. 10.1080/00325481.2016.1147322 26813151

[34] Muhsin SA, Mount DB. Diagnosis and treatment of hypernatremia. Best Pract Res Clin Endocrinol Metab 2016;30:189–203. 10.1016/j.beem.2016.02.014 27156758

[35] Arzhan S, Roumelioti M-E, Litvinovich I et al. Hypernatremia in hospitalized patients: a large population-based study. Kidney360 2022;3:1144–57. 10.34067/KID.0000702022 35919520 PMC9337903

[36] Sterns RH, Rondon-Berrios H, Adrogué HJ et al. Treatment guidelines for hyponatremia: stay the course. Clin J Am Soc Nephrol 2023;19:129–35. 10.2215/CJN.0000000000000244 37379081 PMC10843202

[37] Sterns RH, Cappuccio JD, Silver SM et al. Neurologic sequelae after treatment of severe hyponatremia: a multicenter perspective. J Am Soc Nephrol 1994;4:1522–30. 10.1681/ASN.V481522 8025225

[38] Vu T, Wong R, Hamblin PS et al. Patients presenting with severe hypotonic hyponatremia: etiological factors, assessment, and outcomes. Hosp Pract 2009;37:128–36. 10.3810/hp.2009.12.266 20877181

[39] Grimes DA, Schulz KF. Refining clinical diagnosis with likelihood ratios. Lancet 2005;365:1500–5. 10.1016/S0140-6736(05)66422-7 15850636

[40] Iwagami M, Akazawa M, Ishiguro C et al. Task force report on the validation of diagnosis codes and other outcome definitions in the Japanese receipt data. Jpn J Pharmacoepidemiol 2018;23:95–123.

[41] Rassen JA, Bartels DB, Schneeweiss S et al. Measuring prevalence and incidence of chronic conditions in claims and electronic health record databases. Clin Epidemiol 2019;11:1–15. 10.2147/CLEP.S181242 30588119 PMC6301730

[42] Moder KG, Hurley DL. Fatal hypernatremia from exogenous salt intake: report of a case and review of the literature. Mayo Clin Proc 1990;65:1587–94. 10.1016/S0025-6196(12)62194-6 2255221

[43] Sakamoto A, Hoshino T, Boku K et al. Fatal acute hypernatremia resulting from a massive intake of seasoning soy sauce. Acute Med Surg 2020;7:e555. 10.1002/ams2.555 32832094 PMC7438811

[44] Stelfox HT, Ahmed SB, Khandwala F et al. The epidemiology of intensive care unit-acquired hyponatraemia and hypernatraemia in medical-surgical intensive care units. Crit Care 2008;12:R162. 10.1186/cc7162 19094227 PMC2646327

[45] Arzhan S, Roumelioti M-E, Litvinovich I et al. Outcomes of hospital-acquired hypernatremia. Clin J Am Soc Nephrol 2023;18:1396–407. 10.2215/CJN.0000000000000250 37722368 PMC10637455

[46] Arzhan S, Roumelioti ME, Litvinovich I et al. Hypernatremia in hospitalized patients: a large population-based study. Kidney360 2022;3:1144–57. 10.34067/KID.0000702022 35919520 PMC9337903

[47] Gupta S, Gupta AK, Singh K et al. Are sodium and potassium results on arterial blood gas analyzer equivalent to those on electrolyte analyzer? Indian J Crit Care Med 2016;20:233–7. 10.4103/0972-5229.180044 27303138 PMC4906340

