# Supplementary material for: Diagnostic validity and solute-corrected prevalence for hyponatremia and hypernatremia among 1 813 356 admissions
Source: Clin Kidney J. 2024 Oct 24;17(12):sfae319. doi: 10.1093/ckj/sfae319 (PMC11630772; doi:10.1093/ckj/sfae319)
Supplement: sfae319_Supplemental_File [file sfae319_supplemental_file.pdf]

Supplemental Methods

The equations for corrected sodium concentrations using mmol/L of glucose and triglycerides.

$$\text{Glucose} - \text{corrected sodium concentration} = [\text{Na}^+] + 2.4 \times \frac{glucose \times 18 - 100}{100}$$

$$\text{TG} - \text{corrected sodium concentration} = [\text{Na}^+] + [\text{Na}^+] \times \frac{186 \times TG - 60}{10000}$$

where [Na$^+$] indicates measured sodium concentration, glucose refers to blood glucose concentration (mmol/L) and TG refers to triglyceride concentration (mmol/L)
